# Endocide-Induced Abnormal Growth Forms of Invasive Giant Salvinia (*Salvinia molesta*)

**DOI:** 10.1038/s41598-018-25986-5

**Published:** 2018-05-22

**Authors:** Shiyou Li, Ping Wang, Zushang Su, Emily Lozano, Olivia LaMaster, Jason B. Grogan, Yuhui Weng, Thomas Decker, John Findeisen, Monica McGarrity

**Affiliations:** 1 National Center for Pharmaceutical Crops, Arthur Temple College of Forestry and Agriculture, Stephen F. Austin State University, Nacogdoches, Texas 75962 USA; 2 Inland Fisheries Division, Habitat Conservation Branch, Texas Parks and Wildlife Department, 4200 Smith School Rd., Austin, Texas 78744 USA

## Abstract

Giant salvinia (*Salvinia molesta*) is one of the most noxious invasive species in the world. The fern is known to have primary, secondary, and tertiary growth forms, which are also commonly hypothesized as growth stages. The identification of these forms is primarily based on the size and folding status of the floating leaves. However, we identified 12 forms in the greenhouse and the field. Our experiments showed that the folding of floating leaves is a reversible trait dependent on water access. The floating leaves quickly fold in response to water shortage, reducing water loss and needs, decreasing growth, and avoiding trichome damage. The leaves re-open to allow trichomes repel water and enhance growth when having adequate water supply. Larger secondary or tertiary forms do not produce small-leaf primary forms without high intensity stress. These results do not support the hypothesis that three growth forms represent sequential growth stages. The abnormal small-leaf forms are the result of endocide-induced autotoxicity and some of them never grow into other forms. The development of abnormal forms and reversible leaf folding strategy in response to high stress along with rapid asexual reproduction are major adaptive traits contributing to the invasiveness of *S. molesta*.

## Introduction

Giant salvinia (*Salvinia molesta* D. S. Mitchell; family Salviniaceae), also known as water fern or kariba weed, is native to Brazil. The fern was not recognized as a distinct species from its closely related *S. auriculata* Aubl. until 1972 by D.S. Mitchell^1^. Since 1930, it has gradually become one of the most widespread and environmentally, economically, and socially destructive invasive species^2,3^. Research efforts have been dedicated to *S. molesta*, particularly in elucidate botany^1,4–11^, growth and reproduction^12–19^, ecology and invasion^7,17,20–24^, chemistry^3,25–27^, and control^20–22,26,28–32^. Biological, mechanical, and chemical control measures have been developed for this noxious species yet achieving full control remains a challenge. Salvinia weevil (*Cyrtobagous salviniae*, Curculionidae) was reported to successfully control *S. molesta* in Australia in 1980–1981^31^. However, the weevil is not a cold-tolerant species and salvinia control by weevil is incomplete and dependent upon temeratures and response to stochastic flooding events^2^. Other attempts to manage *S. molesta* through herbicides and mechanical means have failed to achieve full control or eradication^3,24^.

*Salvinia molesta* is a highly invasive, free-floating fern that forms dense colonies of potentially independent ramets^12,33^. Each ramet consists of a pair of floating leaves (fronds), a pendant and highly dissected leaf which functions as a root, an apical bud and usually one lateral bud^17^. New growth arises from these buds. Mature plants have sporocarps but produce infertile spores only because the fern is a sterile pentaploid (2n = 45)^18^. The dense layer of egg-beater glandular trichomes (leaf hairs) on the upper surfaces make the floating leaves extremely water-repellent. The invasiveness of *S. molesta* can be attributed to its ability to spread via fragmentation and its rapid growth. Furthermore, it can regenerate vegetatively even after severe damage or drying for days^17,34^. As a result, *S. molesta* is able to double its biomass in less than three days under optimal conditions and form dense mats over still waters^35^.

It generally accepted that *S. molesta* exhibits three distinct growth forms: “primary-invading form”, “open-water-colonizing-form”, and “mat-form”^13^. The primary-invading form exhibits small, delicate leaves (i.e., <15 mm wide), whereas the leaves of the secondary-colonizing form are slightly cupped and larger (i.e., 20 to 50 mm wide), and in the tertiary-mat form the leaves have reached maximum size (i.e., up to 60 mm wide), are deeply keeled, and have sporocarps^13,15,16,19–21,29^. These three growth forms may appear sequentially during invasion such that some investigators have proposed that there is continuity of growth between them^36^. Some researchers have used phenotypes, forms, stages, or phases to refer these forms, even in the same publications, because these forms acted as the developmental phases^14–16,21^. However, others have described these forms more generally as growth stages^19,25,29^.

Do these three growth forms of *S. molesta* represent sequential developmental stages or rather major phases? Why do primary form plants often emerge after the mass die-offs of *S. molesta*? Why do some primary form plants eventually die whereas others can quickly grow into larger secondary or tertiary forms? Is the development of the smaller, primary form a trade-off in response to high stress? These questions have never been addressed but are critical to understanding the invasion ecology of *S. molesta* and developing an effective control strategy.

## Results

### Are Growth Forms of *S. molesta* also Growth Stages

#### Growth forms of *S. molesta*

**Figure 1:**
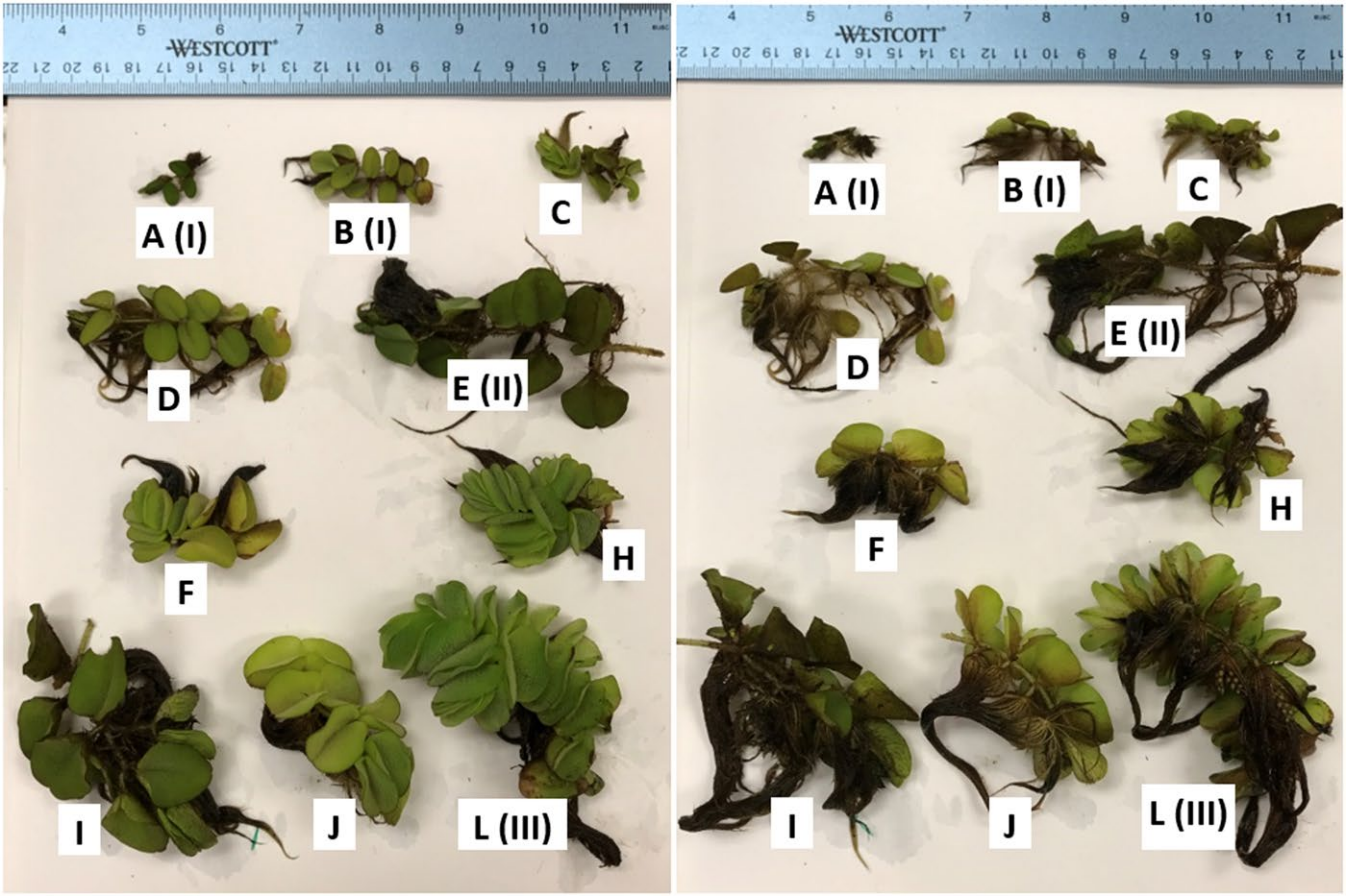
10 of the 12 major growth forms observed in *S. molesta*.

From our observations, there are at least 12 forms that can be identified in *S. molesta* (Supplementary Table S1, Fig. 1), which can be grouped into two major types—normal forms and small abnormal forms. We identified nine normal, large-leaf forms (D-L) and three abnormal, small-leaf forms (A–C). The large-leaf forms include the stages commonly known as secondary (form E) and tertiary (form L). The abnormal, small-leaf forms, include the stage commonly thought to be primary (form A and B). Medium-size normal forms grow fast and can grow up to become mature plants with sporocarps.

#### Impact of population density on growth forms

During the 28 weeks of experimentation, all forms of *S. molesta* experienced population crash following growth peaks. During the entire experimental period, plants of form A at a density of 5 or 15 plants/container produced new growth of form A. Plants of form A at a density of 30 plants/container produced new growth of form A during the first four weeks and then all plants including the new growth turned into form B under these crowded conditions until some plants started dying by the end of six weeks. By the end of the 22^nd^ week, almost all plants cultured at this density died.

Plants of form E at a density of 5 or 15 plants/container produced new growth of form E during the experimentation. Plants of form E at the more crowded density of 30 plants/container produced new growth of form E during the first two weeks and then some began to die. By the end of the 16^th^ week, almost all plants at each density had died out but produced some form A of new growth.

Plants of form L at a density of 5 plants/container produced new growth of form E after growing to fully cover the container by the end of 10 weeks and then started to produce some form L of new growth under crowded conditions. Plants at a density of 15 plants/container primarily produced new growth of form L, and occasionally form E until some original form L plants started dying by the end of the 5^th^ week. After the leaf and stem death of original form L plants at these two densities by the end of the 16^th^ week, form A of new growth appeared. Plants of form L at a density of 30 plants/container produced new growth of form L only during the first six weeks and then produced some form A new growth by the end of the 16^th^ week when the leaves and stems of original form L plants died.

Plants of forms A, E, and L at a density of 5 plants/container generally had higher maximum branch numbers (5, 4.4, and 7.2 branches per plant on average, respectively) than those in any 30 plants/container (4.04, 1.74, and 2.5 branches per plant on average, respectively) (Supplementary Fig. S1). The node number showed a similar pattern as the branch number in the three investigated forms. Plants of the forms A, E, and L at a density of 5 plants/container generally had higher maximum node number (15.27, 11.27, and 43.53 nodes per plant on average, respectively) than those at a density of 30 plants/container (10.52, 5.9, and 12.79 nodes per plant on average, respectively). The result is consistent with the leaf growth. The floating leaf numbers of the forms A, E, and L decreased with increasing density (up to 38.67, 26.27, and 87.07 per plant at a density of 5 plants/container vs. 26.36, 13.81, and 24.33 per plant at a density of 30 plants/container, respectively) (Supplementary Fig. S2). Number of submerged leaves present in forms A, E, and L are usually 50% of the floating leaf numbers and showed the same decreasing trend as floating leaves (up to 19.33, 13.13, and 43.53 per plant at the low density vs. 13.18, 6.91, and 12.04 at the high density, respectively).

In general, each form had the fastest biomass growth at a density of 5 plants/container and the slowest biomass growth at a density of 30 plants/container (Supplementary Fig. S3). The form A, E, and L at density of 5 plants/container had their biomass growth peak by the end of the 16^th^ week and their average plant biomass increased by 668% from the beginning of experimentation (0.57–4.38 g), 251% (1.73–6.07 g), and 886% (3.01–29.69 g), respectively. However, living biomass growth of the form A, E, and L at a density of 30 plants/container reached the growth peak at the 4^th^ week of experimentation and the biomass weight increased only by 345% (0.86–3.83 g), 43% (2.58–3.71), and 88% (2.62–4.92 g), respectively. Also, the plants of each form at the high density went into population recession much earlier than those at the low density.

#### Impact of water accessibility on the folding status of floating leaves

**Figure 2:**
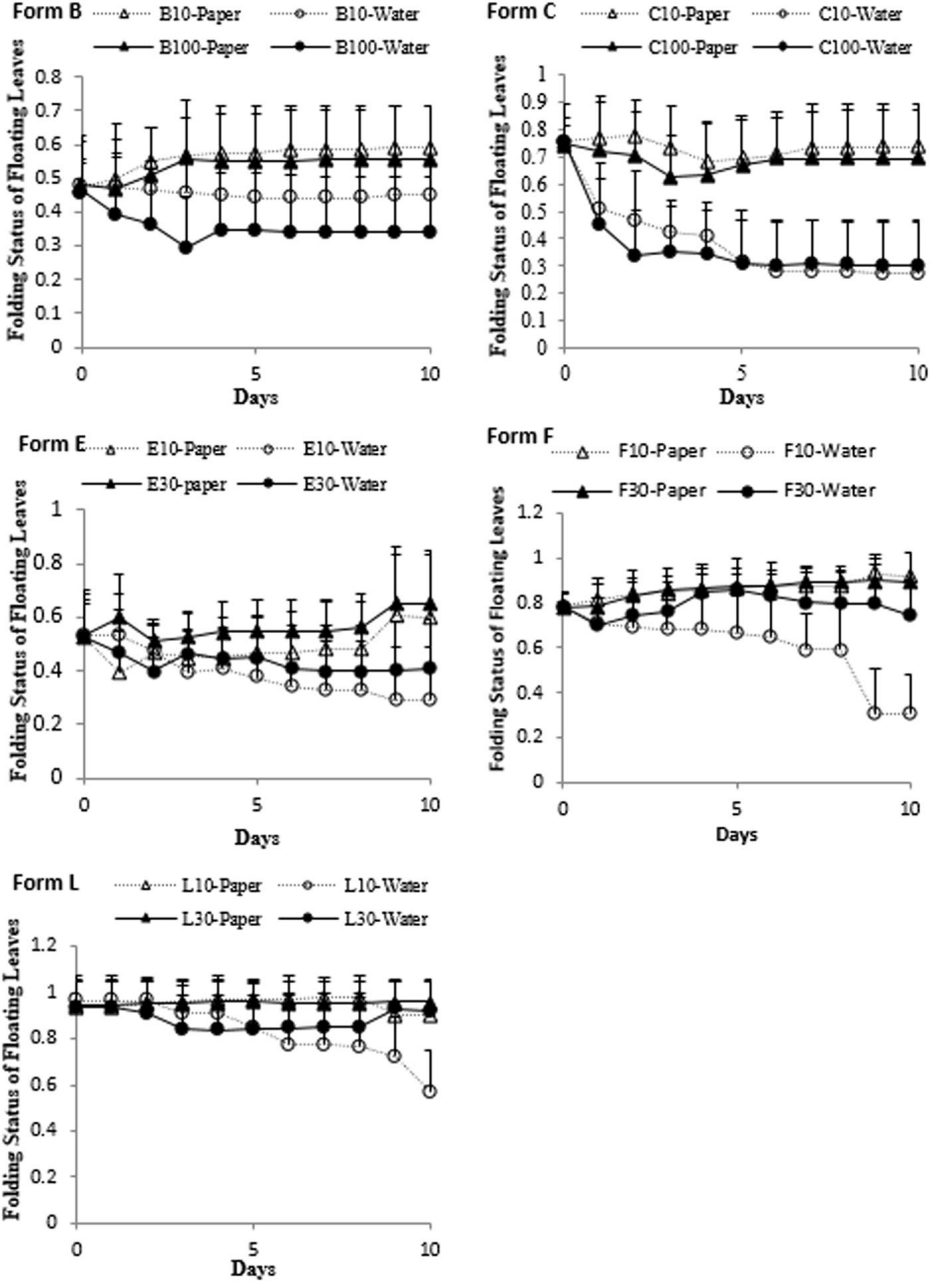
Changes of folding status of floating leaves of forms B, C, E, F, and L on wet paper and in water at different densities during the 10 days of the culture experiment.

As mentioned above, the experimental plants of form B developed from the form C plants after three days of water culture. These form B plants remained as the same form when cultured in water at either low or high density during the 10 days of experimentation (Fig. 2). However, the slightly cupped floating leaves began to fold after 4 h of culture on wet paper towel at either density and transitioned from form B to form C by the end of experiment.

Plants of form C remained having tightly folded floating leaves when cultured on the wet paper towel at either density during the experimentation (Fig. 2). However, their tightly folded floating leaves started to open after 2 h of direct contact with water when cultured in open water at both densities. At the low density, 40–60% of the plants fully opened their leaves within 2 h after direct contact with water and all became form B within 8 h. At the high density, most of the plants opened their floating leaves and transitioned into form B within 24 h and all became form B by the 3^rd^ day of experimentation.

The floating leaves of form E plants became more flat when cultured in water particularly at the low density (Fig. 2). The slightly cupped floating leaves, particularly the young leaves of form E started to slowly fold when cultured on wet paper towel at both densities within 24 h and all new grown floating leaves became noticeably folded close to form F by the end of the experiment.

The folding status of the tightly folded floating leaves of the form F plants became more intensive when cultured on the wet paper towel at either density during the experimentation. Their tightly folded floating leaves started to noticeably open within 2 h when cultured in water at both densities. The new growth from form F plants cultured in water at the low density became form D or E after the 8th day (Fig. 2). When cultured in water at the high density, however, the floating leaves of the form F became more flat during the first couple of days but soon maintained the original folding status as soon as the plants grew too crowded and were then preventing the floating leaves to access the water directly.

The floating leaves of the form L plants kept the folded status when cultured on wet paper towel at either density during the experimentation (Fig. 2). Plants cultured in water at the low density started to open their tightly folded floating leaves within 24 h and become the slightly cupped form K by the end of the experiment. When cultured in water at the high density, interestingly, the floating leaves of form L became more flat after two days but became fully cupped after eight days once they grew to crowded conditions that prevented any floating leaves to contact the water directly.

### How the Small-leaf Primary Forms Arise

#### Impact of fragmentation on growth forms

**Figure 3:**
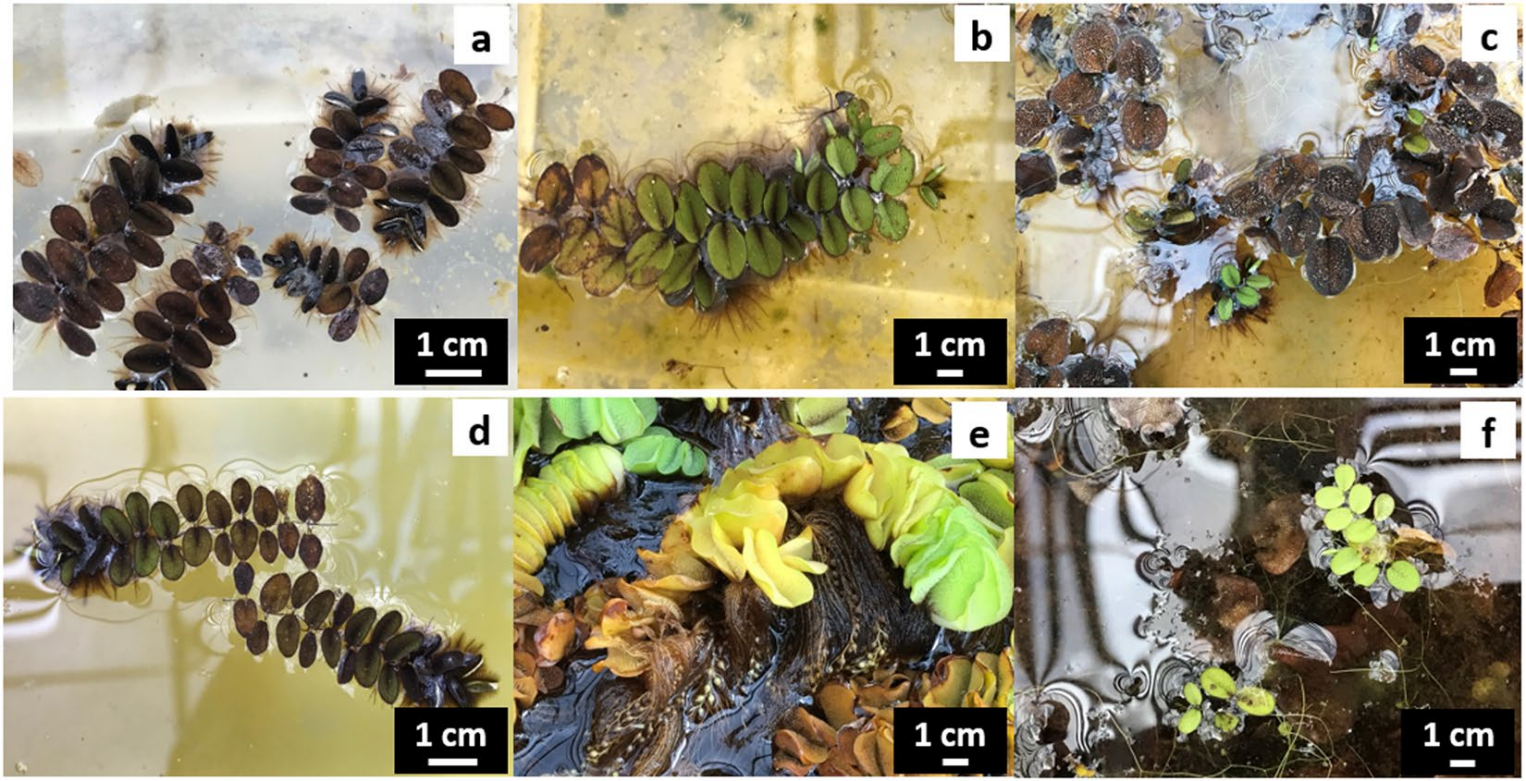
Development of growth forms A, E, and L during the 28 week experiment. (**a**) Dead plants of form A developed from apical buds of form A plant, (**b**) form E new growth developed from an undisturbed colony of form E, (**c**) form A new growth developed from the dead colony of form E, (**d**) form A new growth developed from apical buds of form E, (**e**) form L new growth developed from a undisturbed colony of form L, and (**f**) form A new growth from a dead colony of form L.

Similar to those results in the density experiments, all three forms (A, E, and L) of *S. molesta* with or without fragmenting treatments experienced population crash following the growth peaks during the 28 weeks of experimentation. The colonies without fragmenting treatment, the ramets with apical or lateral buds, or the apical buds of form A developed form A new growth only during experimentation. Some newly developed form A plants were dead after two or three weeks of growth (Fig. 3a) while some others were found to be considered unhealthy. Colonies of form A without any fragmenting treatment and the ramets with apical or lateral buds grew quickly to reach their growth peak at the 12^th^ week of experimentation. Old leaf tissues of the colonies without fragmenting and all initial leaves of the ramets with apical or lateral buds died by end of the 10^th^ week of experimentation. Majority of the form A plants started dying by the 16^th^ week and were almost all dead by the end of the 22^th^ week with a few unhealthy survivors. 8–10 of the apical buds of form A started to grow new leaves during the first week of culture but more 30% of the buds failed to develop any new growth during the experiments. Plants soon died after they reached their growth peak on the 16^th^ week.

Colonies of form E without any fragmenting treatment and the ramets with apical or lateral buds developed form E new growth only in the early stage of experimentation (Fig. 3b). The colonies grew quickly to reach their growth peak after one week of experimentation and the old floating leaves became fully cupped to develop form L status. Old leaf tissues of the colonies without fragmenting and all initial leaves of the ramets with apical or lateral buds died by end of the 10^th^ week of experimentation. All experimental plants of form E started dying after the 12^th^ week and all or almost all died by the end of the 16^th^ week. After leaf and stem death of the form E plants, form B (occasionally form A) new growth was developed from the survived buds (Fig. 3c). Approximately five of the apical buds of form E developed form A or B new growth (Fig. 3d) while only 0–2 lateral buds of form E produced form A or B new growth within the first two weeks. The newly developed form A or B soon died or became unhealthy.

Colonies of form L without any fragmenting treatment developed form E or L new growth (Fig. 3e). The colonies grew quickly to reach their growth peak after one week of experimentation. The ramets with apical or lateral buds of form L developed form E new growth in the early experimental period but some produced form A or B new growth after the initial form L leaves died or their rhizomes broke off from the remaining tissues (Fig. 3f). The living biomass of form L plants with or without fragmenting gradually decreased thereafter, particularly after the 12^th^ week when most plants started dying. About 25% of the apical of form L developed form A or B new growth within the first two weeks of experimentation while only about 8% of the lateral buds of form L developed form A or B new growth during the same time. All newly developed growth was unhealthy and dead by the 28^th^ week.

Colonies without any fragmenting treatment showed slower growth rate in branch, node, floating and submerged leaf numbers in comparison with any of the other treatments. A colony increased by 134–259% in branch number at the growth peak (from the initial one branch to 3.05 during the growth peak for form A, to 2.34 for form E, and to 3.59 for form L) (Supplementary Fig. S4), by 23–195% in node number at the growth peak (from the initial 3.29–9.71 nodes during the growth peak for form A on average, from 5.42–6.66 for form E, and from 8.53–11.56 for form L) (Supplementary Fig. S4), by 26–482% in floating leaf number (4.29–24.96 leaves for form A, 12.58–15.82 leaves for form E, and 14.24–25.47 leaves for form L) (Supplementary Fig. S5), by 26–479% in submerged leaf number (2.14–12.4 leaves for form A, 6.29–7.91 leaves for form E, and 7.12–12.73 leaves for form L) (Supplementary Fig. S5), by 41–480% in total living biomass weight (0.83–4.81 g for form A, 3.02–4.33 g for form E, and 6.81–9.45 g for form L) (Supplementary Fig. S6).

Ramets with apical or lateral buds showed faster growth rate than the colonies without any fragment treatment. A ramet with an apical or lateral bud on average changed from the initial 0 branch to 1.84 or 1.26 branch for form A, to 0.99 or 1 branch for form E, and to 2.29 or 1.80 branches for form L, respectively; from the initial 0 node to 5.92 or 5.17 nodes for form A, to 4.38 or 2.88 nodes for form E, and to 9.26 or 6.82 nodes for form L, respectively; from the initial 2 floating leaves to 13.51 or 11.29 for form A, to 6.5 or 6.39 for form E, and 17.33 or 11.27 for form L, respectively; from the initial one submerged leaf to 6.76 or 5.64 for form A, to 3.25 or 3.19 for form E, and 8.67 or 5.63 for form L, respectively; from the initial 0.22 to 2.50 g for form A ramets with apical buds and from 0.21 to 2.03 g for form A ramets with lateral buds, from the initial 0.50 to 0.82 g for form E ramets with apical buds and from 0.66 to 1.20 g for form E ramets with lateral buds, from the initial 0.47 to 2.51 g for form L ramets with apical buds and from 0.72 to 2.52 g for form L ramets with lateral buds.

Apical buds of form A grew much faster than those of form E or L. Apical buds grew faster than lateral buds for form E and L (no data for form A): on average 4.67 branches per apical bud vs. 1.33 branches per lateral bud for form E and 9.33 vs 1.67 branches for form L; 0.77 node per apical bud vs. 0.17 node per lateral bud for form E and 1.16 vs 0.27 nodes for form L; 1.71 floating leaves per apical bud vs. 0.4 floating leaf per lateral bud for form E and 3.56 vs. 0.53 floating leaves for form L; 0.86 submerged leaf per apical bud vs. 0.19 submerged leaf per lateral bud for form E and 1.78 vs. 0.27 submerged leaf for form L; 0.25 g living biomass per apical bud vs. 0.04 g per lateral bud for form E and 0.74 g vs. 0.32 g living biomass for form L, respectively.

#### Abnormal morphogenesis induced by endocide treatments

In the control treatment (water only), the plants of both form B and E grew during the six weeks of experimentation. The impacts of salvinicide III and IV on *S. molesta* development depend on their treatment concentrations.

All nine plants in the 2% salvinicide III treatment died and showed no new growth during experimentation.

In the 1% salvinicide III treatment container, all six plants of form B or E were dead without new growth during the whole experimental period. By the end of the 2^nd^ week after the 1% salvinicide III treatment, three form A plants emerged from the lateral buds of the form L plants after all the large floating leaves in these form L plants had died or turned brown. However, the three form A plants experienced slow growth and remained in the small and flat form for the following four weeks in the original culture solution. Even after transfer of these three form A plants into another container with new water, these plants failed to turn into any other form during the additional 10 weeks of observation.

**Figure 4:**
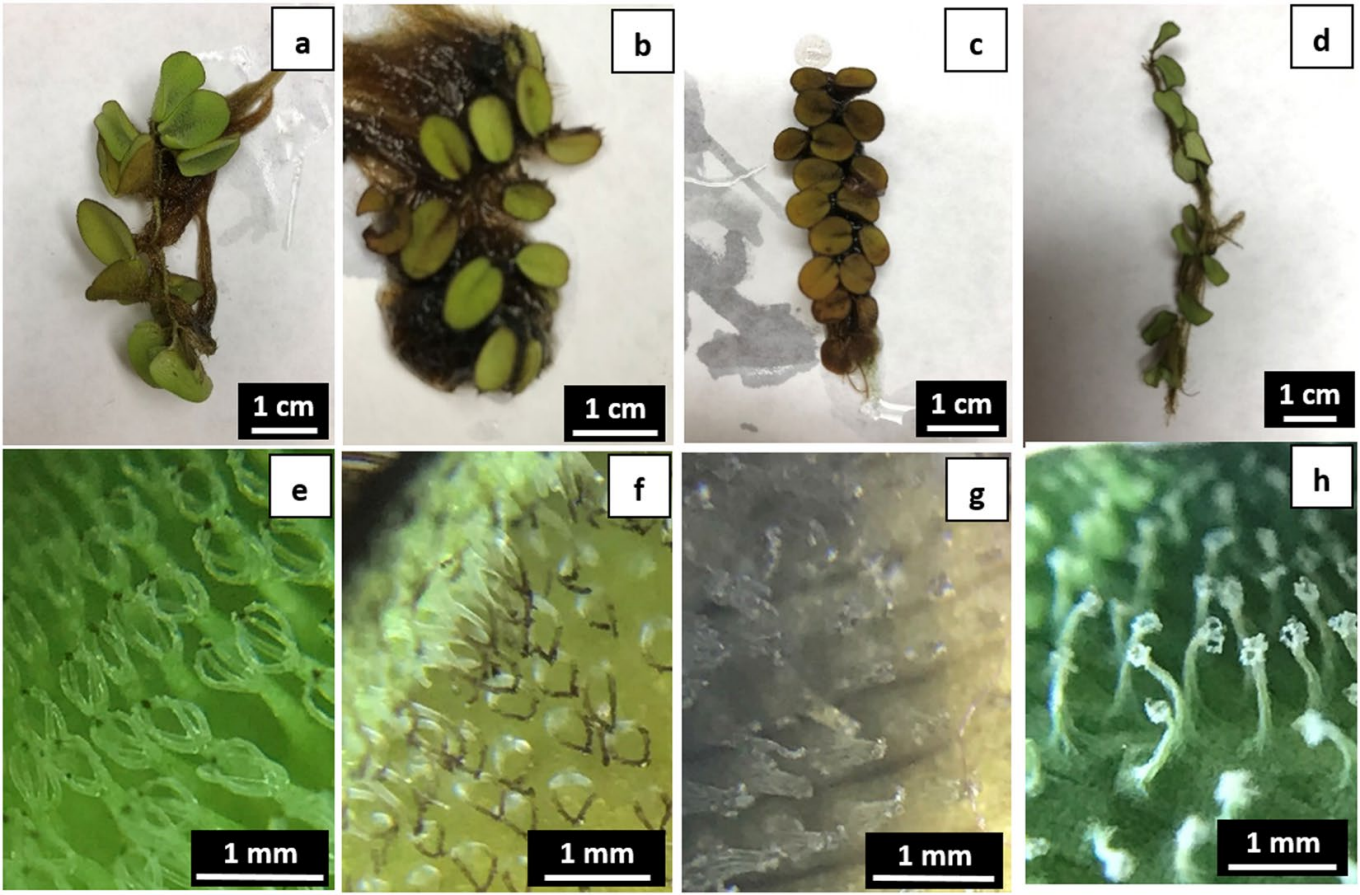
Healthy and unhealthy abnormal small-leaf growth forms of *S. molesta* induced by endocide. (**a**) and (**e**) a healthy plant of form B with normal trichomes would grow quickly into a large-leaf form; (**b**) and (**f**) an unhealthy plant of form A with damaged heads of trichomes would die soon without detoxification; (**c**) and (**g**) a plant of form A dying because of endocide released from the damaged trichomes; and (**d**) and (**h**) a healthy plant of form A died due to drying but the remained their undamaged trichomes.

The application of 0.5% salvinicide III killed almost all floating leaves of the nine treated plants within a week. By the end of the 2^nd^ week, new floating leaves had emerged from eight plants and one form B plant had died. During the six weeks of experimentation, only one form L plant with partial green apical floating leaves developed as form B then as form E new growth. According to our observation, this form B plant was healthy and its trichomes remained intact (Fig. 4a,e). During the same period, however, all other newly emerged plants from lateral buds remained as form A. These small plants were not healthy and all trichome heads had significant damages at the beginning (Fig. 4b,e). In the original culture solution, some of these plants lost their trichome heads and died later (Fig. 4c,g). The surviving plants did not obviously improve their conditions even after transferring into new containers with new water.

The 0.25% salvinicide III application killed all floating leaves of the form B or E plants and partially injured floating leaves of the form L plants during the first week. Seven plants developed form E new growth from apical buds of six form E or L plants and one form B plant by the end of the six weeks of experimentation. Two emerged plants from the form B plants remained in the same form during the whole experimental period in the original culture solution. The number and size of floating leaves and submerged root-like leaves and internode length of all nine plants increased after transferred into a new container.

By the end of experimentation, the *S. molesta* plants treated by 0.125% or lower concentrations had no significant damage. Similar to those in the control group, there were only form L plants observed in these containers by the end of the six weeks of experimentation.

Similar to salvinicide III, salvinicide IV (0.25, 0.5, and 1%) also induced small leaf mutations in *S. molesta*. More new growth was observed following the treatments of 0.5% or 1% salvinicide IV than the treatments of salvinicide IV, but the new plants remained as form A during the six weeks of observation. 2% salvinicide IV treatment killed all of the plants and no new growth was observed.

#### Trichome damage

**Figure 5:**
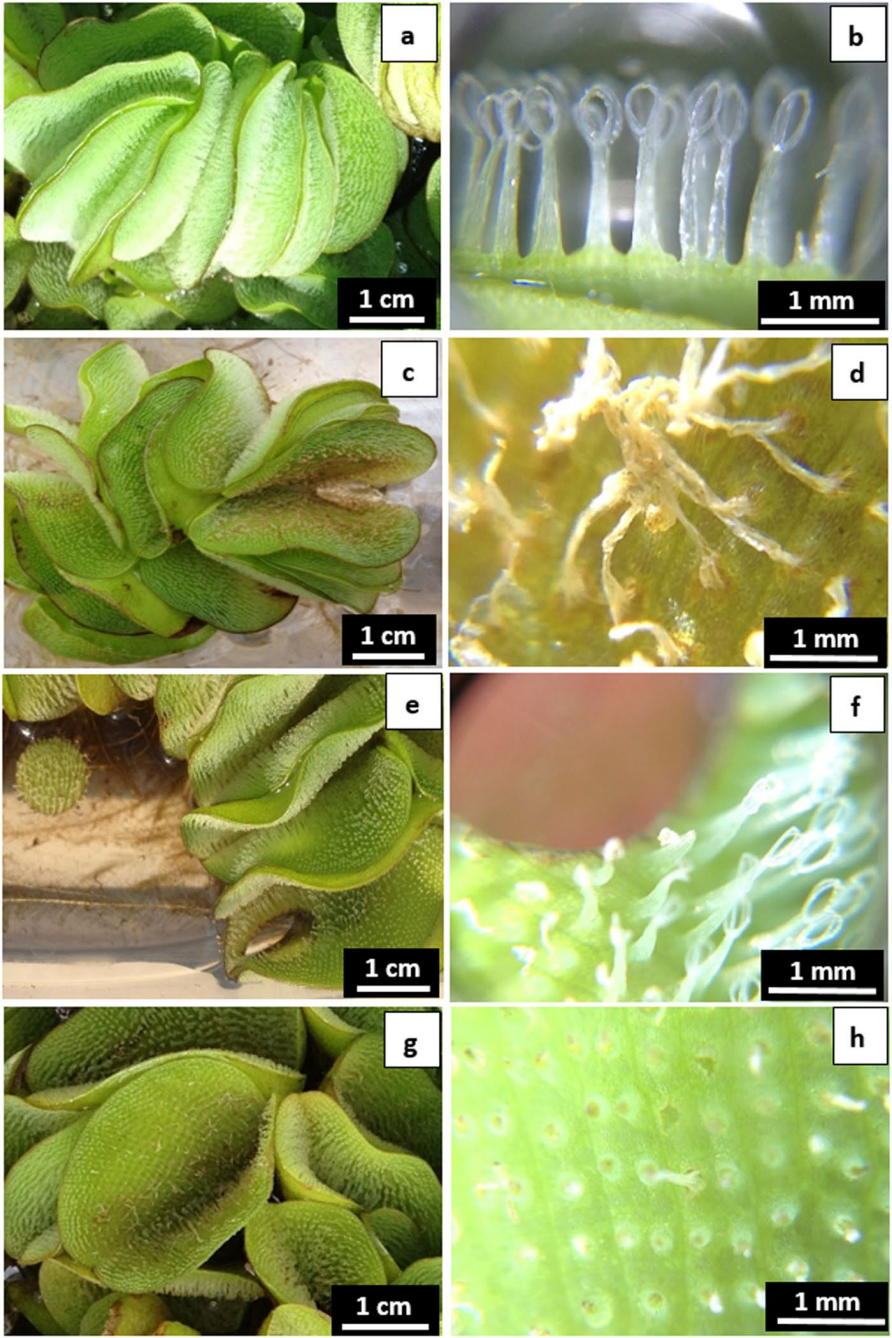
Damages of three different physical damages on floating leaves of *S. molesta* 4 days after the treatments. (**a**) and (**b**) floating leaves and trichomes without a disturbance served as controls; (**c**) and (**d**) floating leaves and trichomes were impacted by upper surface rubbing because the rubbing released endocides from damaged trichomes to the surface and thus caused leaf surface tissue damage; (**e**) and (**f**) floating leaves and trichomes were not impacted by hole punching in leaf blade; and (**g**) and (**h**) floating leaves and trichomes were not impacted by removal of trichomes without residues left on the leaf surface.

**Figure 6:**
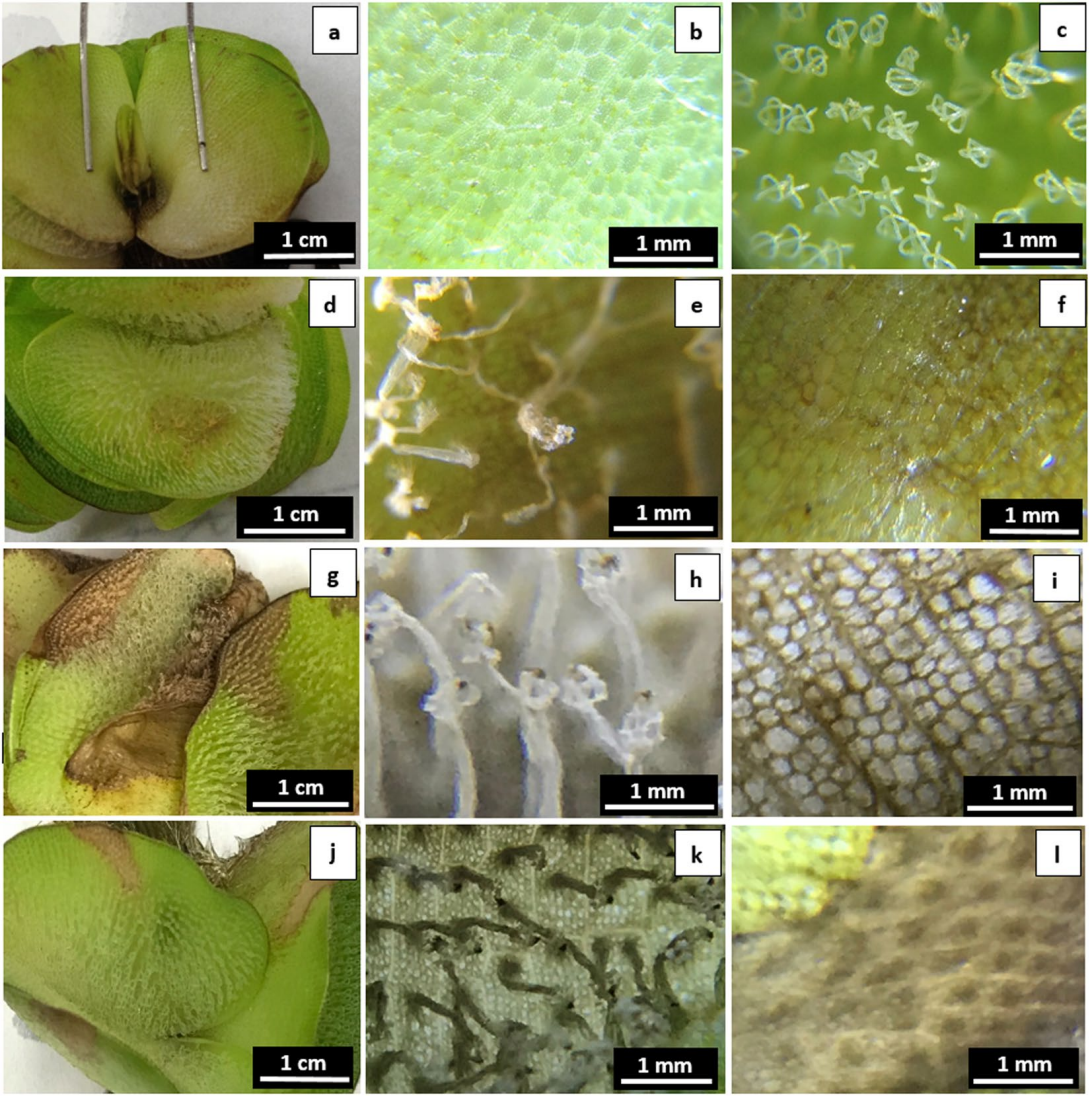
Effects of Dyne Amic® and salvinicide III on lower and upper surfaces of floating leaves of *S. molesta* four days after the treatments. Application of 8 µL 0.5% Dyne Amic® on lower leaf surface did not cause any damage on *S. molesta* (**a**–**c**) but application of the same dosage on upper leaf surface caused significant leaf damage (**d**–**f**). Application of 8 µL 0.5% salvinicide III on either lower (**g**–**i**) or upper leaf surface (**j**–**l**) cause significant damage on *S. molesta* leaves.

Among the three physical treatments on floating leaves of *S. molesta*, only rubbing caused damages on leaf surface tissues four days after the treatment (Fig. 5). Rubbing immediately destroyed the trichomes while the basal cells of the trichomes turned brownish on the four days after the treatment. The whole leaf blade in the area with trichome rubbing turned brownish by the end of three weeks. However, the leaves had no observable damages following either hole-punching or trichome removal during the three weeks of experimentation. Dyne-Amic® did not show phytotoxicity on *S. molesta* because its application (at concentration of 0.5%) on lower leaf surface did not cause any damage, but its application on upper leaf surface damaged some leaf tissues after it wilted trichomes (Fig. 6). 0.5% Salvinicide III destroyed the leaf tissues when applied on either lower or upper leaf surfaces (Fig. 6).

## Discussion

### The Growth Forms of *S. molesta* are the Result of Environmental Impacts not Growth Stages

#### Leaf Water Accessibility rather than Population Density Determines the Folding Status of Floating Leaves

It has been commonly recognized that the following relationship is present between the growth forms (stages) and density^16,21,29^: primary growth stage (form A or B) occurs in adverse conditions or during the initial invading stage of the infestation and thus the population density is low; the secondary stage is found in open water (either freely or on the edge of stable mats); and the tertiary stage (form L) occurs only under higher density. It seems that population density or crowding condition is the primary cause of the growth stages (forms) in *S. molesta*. The classification of these growth forms (stages) of *S. molesta* is basically based on the folding status of floating leaves.

The present experiments showed that the tightly folding leaf forms C, F, and L remained the same folding leaf status when cultured in wet paper towel at either low density (10 plants/container) or high density (100 or 30 plants/container). When cultured in water at low density, the tightly folded floating leaves of these forms were able to fully or partially contact water directly and thus became open as the slightly cupped form B, E (D), or K within 10 days, respectively.

When cultured in water at higher densities, the forms C, F, and L developed differently. The plants of form C remained as form B after three days of culture in water because their floating leaves could fully or partially contact water directly. However, the floating leaves of the forms F and L mainly maintained their tightly folding status during the whole experimental period because all floating leaves were not able to directly contact water.

In contrast, the slightly cupped leaves of form B or E remained in the same form when cultured in water at either density. The form B or E became more folded after several days of culture on wet paper towel at either low or high density. Because at the same density, these forms developed differently when cultured in water and wet paper towel, density is not a primary factor for folding status of floating leaves.

**Figure 7:**
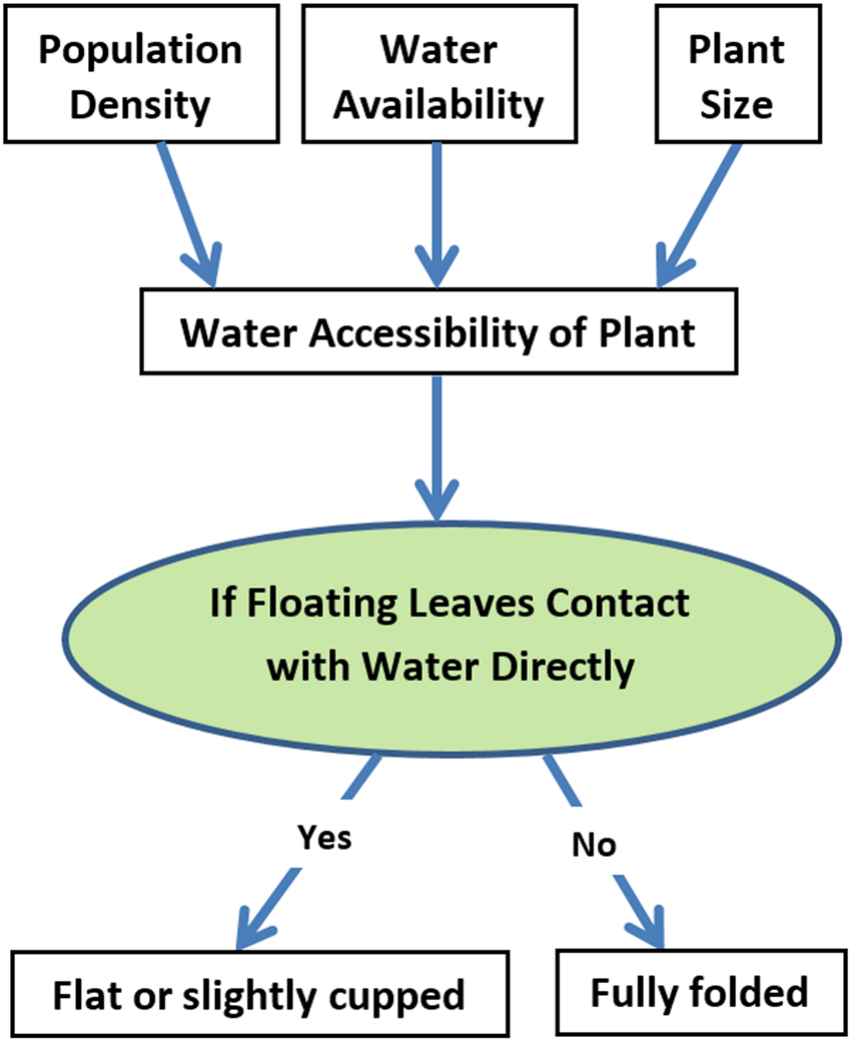
The folding status of floating leaves of *S. molesta* is primarily determined by if the leaves are able to contact the water directly. The water accessibility of the floating leaves are impacted by some secondary factors such as population density, plant size, and water availability.

Our data lead to the conclusion that the folding status of floating leaves of *S. molesta* is primarily determined by direct water contact of the leaf. Population density or crowded conditions, plant size, and water availability impact the folding status of floating leaves via theirs impact on water accessibility. Once the floating leaves fully contact water, it will display flat or slightly cupped status (Fig. 7). If only submerged root-like dissected leaves contact water, the floating leaves will become tightly folded. High dense population or crowded condition may prevent floating leaves of some plants from contacting water directly and thus the plants developed or remained folded floating leaves only.

The experiment showed that increasing density generally reduced branching of *S. molesta*. This result is consistent with the previous observations^12,13,16,19^.

#### Plasticity and Reversibility of Leaf Folding Status with Water Accessibility

**Figure 8:**
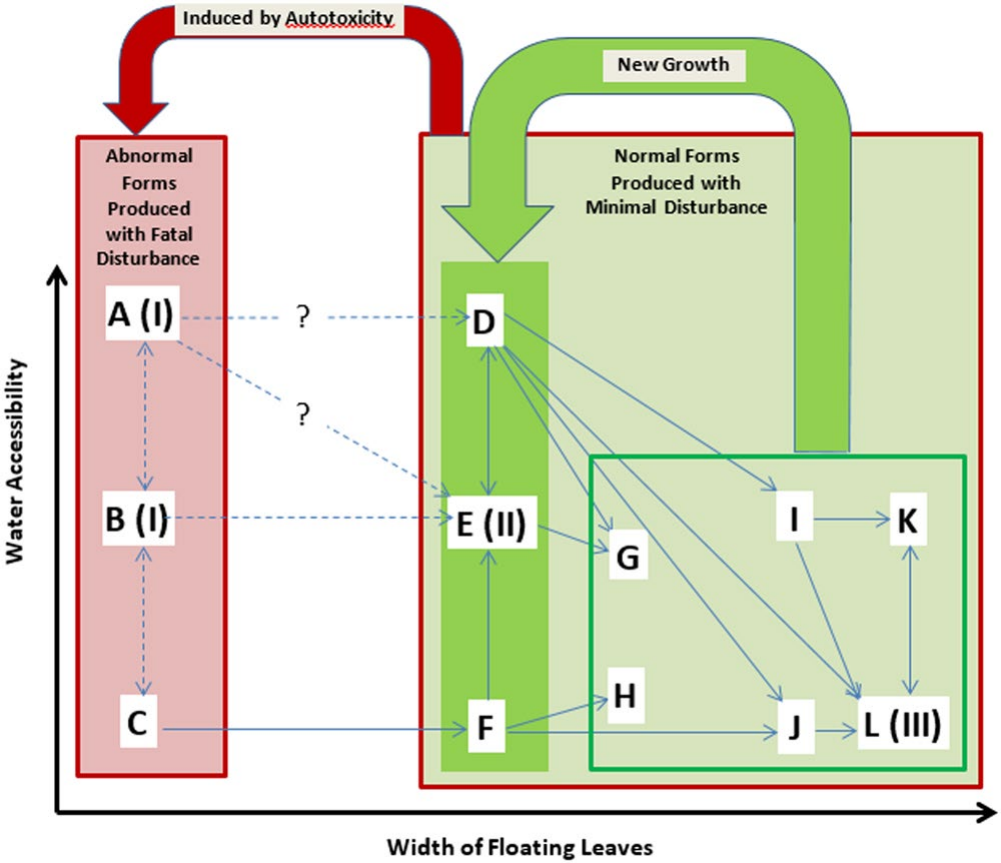
There are primarily two different types of growth forms of *S. molesta* that may develop: normal forms (D–L) under minimal disturbance and small abnormal forms (A–C). Medium-size normal forms grow fast and can grow up to become mature plants with sporocarps. Abnormal forms are developed from normal forms induced by autotoxicity following fatal disturbances such as extensive herbicide, weevil, or endocide treatments, physical damage, extreme climatic conditions (e.g., drought and freeze). Abnormal form may die out without further development or develop forms (D), (E), or F under certain antidotal conditions (e.g., flowing water and mixed population with other plant species) (see dash arrowed lines). Growth space available is an important factor that determines growth forms. The same plants can grow as A or D when adequate space available, or B or E when limited space available or C or F under crowded conditions.

As demonstrated in the experiments, floating leaves (fronds) of *S. molesta* exhibited reversible change between slightly cupped and tightly folded statuses in all growth “stages”: young (form B and C), fast growing (E and F), and mature stages (K and L) (Fig. 8). The floating leaves would be slightly cupped if they directly contact water but become tightly folded when they lost direct contact with water. When grown freely (e.g., initial invading stage of infestation or in low density) or on the edge of stable mats, plants have slightly cupped or flat floating leaves, presumably because the leaves can contact the water directly. However, the leaves can vary with growth age: newly developed leaves are small (form A or B), then some become larger in size (form E or I) without sporocarps, and finally develop sporocarps at maturity (form G or K).

When grown on soils or occur under conditions that the floating leaves are not able to contact water directly, such as on the top of other plants or crowded conditions (e.g., late infestation stage), plants remain tightly folded (deeply keeled) due to limited space throughout the growth period and the new growth are small floating leaves (form C), then become larger in size (form F or J) without sporocarps, and finally develop sporocarps at maturity (form H or L).

Because the folding status of floating leaves exhibits plasticity and reversibility with water accessibility, it should not considered as a primary factor to determine growth stage or type. Morphological plasticity has been reported in other *Salvinia* species: *S. auriculata* plants under densely crowded conditions were significantly larger than plants under uncrowded conditions^37^.

The plasticity and reversibility of leaf folding is a strategy of *S. molesta* adpated to dynamic water supply. In response to water shortage, floating leaves can quickly fold. The folded leaves decrease surface area exposed to sunlight and thus reduce transpiration loss and water needs. It also slows plant growth by decreasing leaf area for photosynthesis. The low demands strategy helps *S. molesta* to withstand high stress environment. The opening of floating leaves in response to adequate water access will not only allow the trichomes to repel water but also increase leaf area for photosynthesis. Therefore, leaf opening is a plant strategy to stimulate growth in a favorable environment.

#### Secondary and Tertiary Forms of *S. molesta* Develop A Small-leaf Primary Form only after Fatal Damages

During the experimentation, form A plants of *S. molesta*, no matter whether they are damaged or not, produced form A new growth only. Some newly developed form A plants died dead after two or three weeks of growth and had no chance to grow into other forms. However, form E or L plants developed either form E or L new growth upon water accessibility but did not produce form A, B, or C new growth unless damaged or dead. As Oliver stated that *S. molesta* in a new habitat develops the colonizing stage (form E) plants with thin stems and fragment easily to produce new plants^29^. In our experiments, undisturbed colonies or fragmented ramets of form E produced form E new growth when the new floating leaves were able to fully contact water or developed into form L plants or directly produced form L new growth when the new leaves were not able to fully contact water directly. Undisturbed colonies of form L could develop either form E or L new growth while damaged form L ramets usually develop form E new growth but produce form A or B new growth after the initial plants died or their rhizomes broke.

### Small-leaf Primary Forms of *S. molesta* are Abnormal Growth Forms Induced by Autotoxicity

#### Small-leaf Primary Forms due to Fragmentation

Without fragmentation, form A plants of *S. molesta* grew faster in number of node, floating, and submerged leaves and weight of living biomass than either form E or L. For example, the living biomass of form A colonies could increase by 480% (0.83–4.81 g) during the 28 week of experimentation while that of form L increased by less than 39% (6.81–9.45 g) during the same period (Supplementary Fig. S6). However, the growth advantage of form A was not found in the fragmented plants. The fragmented ramets of form A (with or without apical buds) had similar slow growth rates with those of the form E or L. This is because all forms could be poisoned by autotoxicity due to fragmenting. This investigation did not characterize the endocidal changes after fragmenting because the analytical HPLC (high performance liquid chromatography) was not sensitive enough to detect the salvinicide changes of small samples of *S. molesta*. We expect the *S. molesta* experience similar endocidal enhancement following pruning as reported in *Camptotheca acuminata* Decaisne (Nyssaceae)^38,39^. Further, small-leaf forms may contain higher levels of endocides and maybe more susceptible to induced autotoxicity than the large-leaf forms. This may partly explain why some small-leaf forms never grow into other forms and even die out without noticeable cause.

Our experiments showed that larger-leaf forms (D–L) of *S. molesta* would not produce any small-leaf forms (A–C) unless they experienced severe or fatal damages. This result is consistent with the previous observation by some authors that primary stage is the result of recovering from damage or in uncrowded conditions in shade or rich nutrient culture^9,21^. Although the small-leaf forms often occur in adverse conditions or during the initial invading stage of the infestation^16,21,29^, primary form plants are not necessarily in the early development stage. They may be produced following damages in the large-leaf forms or fragmented directly from small-leaf plants. Some of these small-leaf plants may die out before growing into other forms. Thus, commonly known primary, secondary, and tertiary forms are not sequential growth stages.

#### Small-leaf Primary Forms as the abnormal morphogenesis Induced by External Application of Endocides

Our results indicated that the small-leaf forms can be induced by direct application of endocides (salvinicide III and IV) treatments of the large-leaf form plants. In *S. molesta*, the abnormal forms A-C are the result of endocide-induced autotoxicity. Previously we reported that similar small-leaf mutations caused by autotoxicity in flowering plants such as *Camptotheca* Decaisne and Chinese tallow (*Triadica sebifera* (L.) Small) (Euphorbiaceae)^26,39^. Small leaf cultivars were developed from *C. lowreyana* by extended seed soaking in water while small leaves developed following the treatment of glyphosates or decapitation pruning in *T. sebifera*^26^.

#### Trichomes play a critical role in the endocidal regulation

On the upper surfaces of the floating leaves of *S. molesta*, there are multicellular glandular trichomes have their apical cells connected to form egg-beater structures. We reported that endocides of *S. molesta* are primarily accumulated in trichomes which are usually intact so that the plant can avoid poison^26^. The endocides in intact trichomes of the living plants are not available the surrounding water at substantial amount because of the water-repellent feature of trichomes. However, the damage of the apical cells of trichomes will not only release the chemicals in storage but also allow water to access the floating leaves to dissolve the bioactive agents. This explains why physical (e.g., rubbing) or chemical damage (e.g., non-phytotoxic Dyne Amic®) can damage the leaf surface tissues by endocide release but cut tissue off or removal of trichomes without residues on the leaf surface does not impact the leaf tissues. Clearly, status of trichomes on the upper surfaces of floating leaves determines if the abnormal small *S. molesta* plants further develop. The folding of floating leaves in response to water shortage is a plant strategy to prevent glandular trichome damages and to avoid endocide-induced autotoxicity In practice, any methods for breaking the trichomes (e.g., mechanical treatments such as grinding, blending, squeezing, or heavily washing plant tissues) is necessary for effective extraction of the bioactive agents from *S. molesta*.

Salvinia autotoxicity is usually induced by damage or fragmentation caused by stresses such as physical factors (e.g., freeze, winds), biological factors (e.g., herbivory by insects including weevils), or chemical factors (e.g., herbicides, endocides, and surfactants). The damage or fragmentation may directly induce the endocidal enhancement in the plants and the endocidal induction results in the mutations in the plants. In *S. molesta*, the endocidal compounds may include salvinicides III and IV and the previously reported salviniside II and ( + )−3-hydroxy-*β*-ionone^26^. Because the parent species produces and uses these endocides in regulation of its physiological processes, its cell plasma membrane and/or wall (e.g., secondary cell wall) may be selectively permeable to these metabolites. As a result, the producing species are more susceptible to their own metabolites than to external agents. The quick response of *S. molesta* and other plants and insects to the endocides within several hours in lab/greenhouse/field tests supports the hypothesis^26^. This possible mechanism also explains why endocides are usually more toxic to the parent species than non-closely-related species.

#### Small-leaf Primary Forms as the Key of *S. molesta* Control

**Figure 9:**
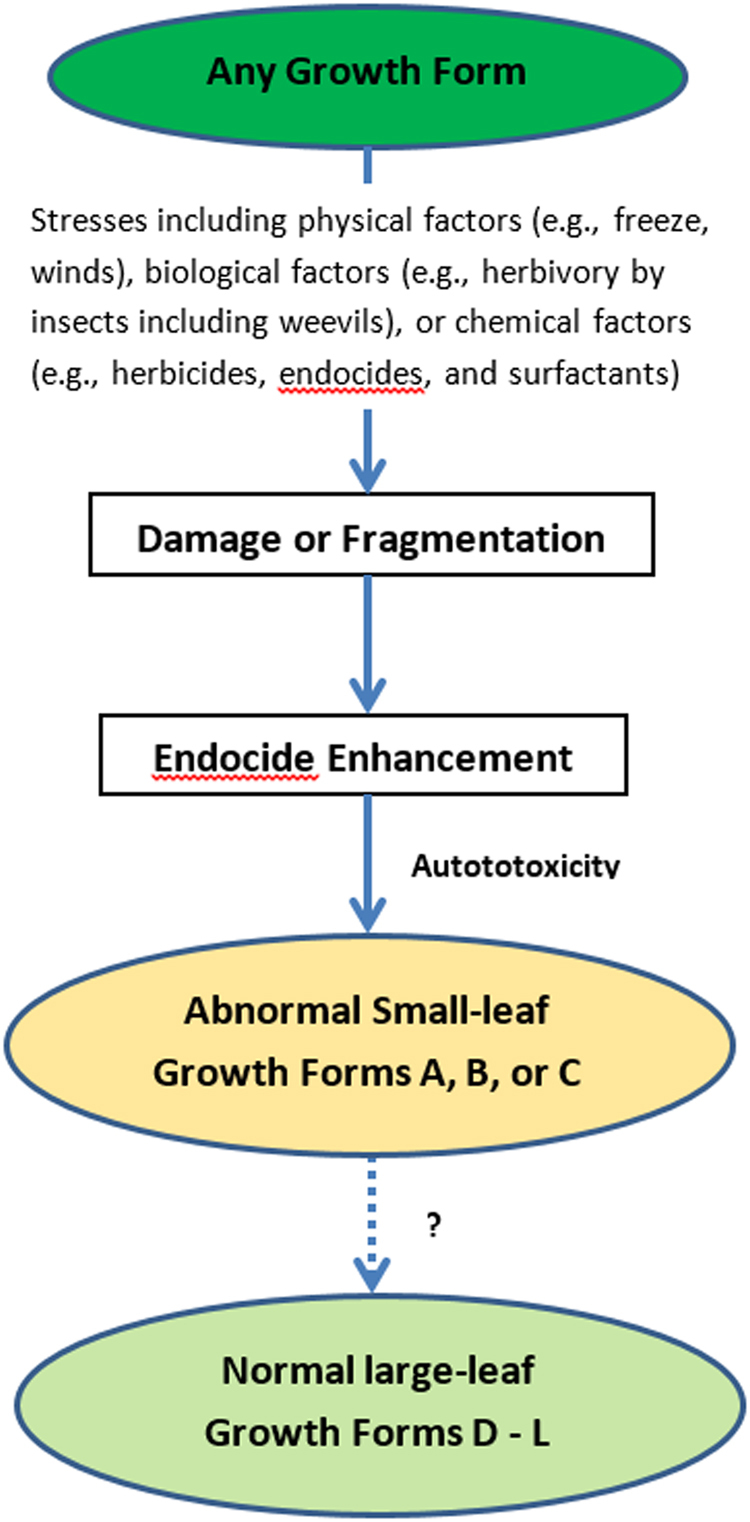
The abnormal small-leaf forms, including forms A (known as primary stage), B, and C, are the mutations due to endocide-induced autotoxicity caused by damage or fragmentation of any growth forms of *S. molesta*. Whether the abnormal forms develop to normal forms is uncertain and the causes remain elusive.

From our observations, there are at least 12 forms that can be identified in *S. molesta*, including nine normal large-leaf forms and three abnormal small-leaf forms (Supplementary Table S1 and Fig. 1). The commonly known forms (primary, secondary, and tertiary stages or phases) are just three of the 12 observed forms in *S. molesta*. The large-leaf forms D to L, including the known secondary stage (form E) and tertiary stage (form L) are normal type of growth forms that can be found under the conditions with minimal disturbance. These forms are commonly found in establishment stage, particularly when they are not treated or disturbed. However, these plants can be easily controlled by herbicides and these forms may even seasonally appear in regions with cold winter temperatures. The abnormal small-leaf forms, including forms A (known as primary stage), B, and C, are the mutations due to endocide-induced autotoxicity (Fig. 9).

The large-leaf forms (D–L) may dominate the *S. molesta* infestation in the water body in terms of both space and biomass. These plants can be knocked down by many control measures, cold weather, or naturally die after they reach maturity. However, *S. molesta* infestation seems to be a recurring problem. Following the death or damage of the large-leaf form plants, small-leaf forms (A–C) may develop. According to our greenhouse and field observations, as well as our experiments, these forms can be developed from large-leaf forms when their buds experience autotoxicity. If buds experience extensive and prolonged autotoxicity, for example, enhanced production of endocides due to critical damage as apical buds removed from the terminals of the colonies or direct application of endocides), these forms remain small and unhealthy and eventually die. If buds experience short or less extensive autotoxicity, these forms can still grow up to become large-leaf forms. Form C may directly develop form B due limited water accessibility and if it occurs under crowded conditions or on soil. Thus, the elimination of *S. molesta* in an area by a control measure is largely determined by the development of small-leaf forms.

It is not clear under what conditions this abnormal form A or B would die out or grow into large-leaf forms, but plants with intact trichomes will likely have further development. Likely these abnormal plants will soon grow into normal forms under antidotal condition such as flowing water or mixture with other plants. In *Camptotheca*, it was found that auxins are a triggering factor causing the restoration of dwarf ‘CT168’ to a normal phenotype^39^. Future investigation needs to elucidate role of auxins in the induction, development, and restoration of the *S. molesta* small-leaf form mutations because the understanding of this mechanism is critical to the control of this noxious invasive species.

The trade-offs to grow, survive, and reproduce in response to environmental variations are common and thus plants develop various strategies that demonstrate resource trade-offs between growth, reproduction, and maintenance when responding to varying levels of disturbance and stress^40^. J.P. Grime characterized these strategies as competitors (C), stress tolerators (S), and ruderals (R)^40^. According to this CSR triangle hypothesis, plants are not viable in an environment with high intensity of disturbance and high stress. The hypotheses or the universal adaptive strategy theory was developed based on terrestrial plants that reproduce sexually. *Salvinia molesta* is an aquatic species that reproduces vegetatively. It can grow and reproduce very quickly and becomes a dominating species in a low stress and low disturbance environment. In this case, it acts like a competitor in the Grime’s classification. However, *S. molesta* can quickly colonize open waters with high intensity of disturbance and low intensity stress and thus dominate the habitat for years like a ruderal species. Although *S. molesta* is tolerant of stress, unlike a terrestrial tolerator it exhibits high phenotypic plasticity under high environmental stress. The plant produces the abnormal small-leaf forms following either high stress (e.g., fragmentation) or high disturbance (e.g., cold damage, chemical applications). Although some from both environments will eventually develop into large-leaf plants, many small-leaf from plants grow very slowly and remain small leaf forms for a long period of time while some may even die under continuous high stress conditions. The small-leaf forms may be advantagous over large-leaf forms in enabling the plant to adapt to the harsh environment by reducing water and other resource needs. In response to limited water access, *S. molesta* can quickly fold its floating leaves, thus reducing water loss and needs and avoiding glandular trichome damages which result in endocide release to induce autotoxicity. When the floating leaves have adequate water access, these leaves will re-open to repel water and increase photosynthesis performance. The development of abnormal small-leaf forms and reversible leaf folding strategy of *S. molesta* in high-stress environments along with rapid asexual reproduction are major adaptive traits allowing it become one of the most invasive plant species in the world.

## Materials and Methods

### Plant materials

By using the classification characteristics to distinguish the known three stages (forms) of *S. molesta*, we have actually identified 12 different forms of *S. molesta* in the greenhouse or the field (Fig. 1, Supplementary Information 1 and Table S1). Only forms A, B, C, E, F, and L were included in the experimentation. Experimental plants of form A (commonly referred to as primary stage; I) resulted from low concentration endocide treatments in the greenhouse (30 °C during the day and 20 °C at night). Experimental form C plants were collected from the shoreline (i.e., on soil) of Sam Rayburn Lake, Texas, USA in early May 2017. Approximately 800 plants of form C were cultured in plastic containers (60 × 43 × 15 cm each) with 15 L of tap water. Three days later, 100% of the tightly folded floating leaves of the form C plants were open to become form B (sometimes also referred to as primary stage; I). Plants of form E (secondary stage; II) were collected from the open water surface of Sam Rayburn Lake, Texas, USA in early May 2017. Experimental form F plants were selected from cultured plants which were initially collected from Sam Rayburn Lake, Texas, USA in June 2016. Experimental plants of form L (tertiary stage; III) developed from the cultured plants in the greenhouse which were initially collected from Sam Rayburn Lake, Texas, USA in June 2016.

For each form, only healthy colonies containing 4–6 pairs of floating leaves without any side branches were selected to start the experiments. These plants were “similar to the 4-node “standard plants” of Room (1983)^14^ and Room and Thomas (1986)^17^ or “3-node plants” of Solangaarachchi and Hapuarachchi (1995)^16^. In the density and leaf folding experiments, the experimental plants of form A, B, or C weighed 0.3 g each, the form E plants weighed 2.0 g each, the form F plants weighed 2.2 g each, and the form L plants weighed 3.9 g each on average.

### Experimental endocides

The experimental endocides salvinicide III (>95%, HPLC analysis) and IV (>95%) were isolated from *S. molesta* matter by using the method described by Li *et al*.^3^. Salvinicides III and IV were prepared as 50 mL experimental solution with Nanopure™ water at eight concentrations, 0.015, 0.031, 0.063, 0.125, 0.25, 0.5, 1, and 2%, respectively.

### Density experiments

Three different densities of each of the forms A, E, and L were investigated. For each form, 5, 15, or 30 colonies that each container 4–6 nodes were cultured in a plastic container (23 × 23 × 6.8 cm) with 2.35 L of tap water. The same water level was maintained for each container during the experimental period. There were three replicates in each of the three density experiments. The experiments were started on November 30, 2016. The numbers of branches, nodes, floating leaves, and submerged leaves, and biomass weight of the plants in each container were measured on week 0, 1, 2, 3, 4, 5, 6, 8, 10, 12, 16, 22, and 28.

### Floating leaf folding experiments

The experiment included forms B, C, E, F, and L. The forms C and E in the leaf folding experiments were collected from the field directly two hours before the experiments. Form B plants were developed from water culture of form C after three days. Others were selected from the greenhouse culture before the experiments. Each of the selected plants contained four nodes. To manipulate the two major natural habitats of *S. molesta* (open water and soils), each form was investigated for its folding change of floating leaves at two densities in plastic containers (23 × 23 × 6.8 cm each) under one of two culture conditions. The low density was 10 plants per container and the high density was 100 plants per container for forms B and C and 30 for forms E, F, and L. The two culture conditions included the containers filled with 2.35 L of tap water or 0.2 L of tap water (adequate to saturate the three layers of paper towel but no visible water layer on the towel. The water in each container was remained at the same level during the experiment period. There were three replicates each treatment. The experiments were started on May 23, 2017. The plant growth of each treatment was photographed daily for 10 days. During the period, the overall folding status of each plant was recorded daily as the average number of the folding status of all floating leaves while actual folding status of each individual leaf was estimated by an index number between 0 and 1.0. For example, 0, 0.25, 0.5, 0.75, and 1.0 represents the flat, slightly folded, folded, tightly folded, and totally folded status of a pair of floating leaves, respectively (the angle between the upper leaf surfaces of the pair is 180°, 135°, 90°, 45°, and 0°, respectively). Therefore, the *S. molesta* growth forms were defined by the folding index as follows: forms A and D <0.25; forms B, E, G, I, and K: 0.25–0.75; and forms C, F, H, J, and L >0.75).

### Fragmentation experiments

The forms A, E, and L, representing commonly known growth stages (I, II, and III, respectively) were tested in the experiments. The five treatments included (1) intact colonies with 4–6 nodes naturally each had no treatment (control); (2) single node cuttings (ramets) with a pair of floating leaves, a submerged dissected leaves, and apical buds; (3) single node cuttings (ramets) with a pair of floating leaves, a submerged dissected leaves, and lateral buds; (4) apical buds; and (5) lateral buds. For each treatment, 30 experimental colonies, ramets, or buds were cultured in a plastic container (60 × 43 × 15 cm) in which 15 L of tap water was added and the same water level was maintained during the experimentation. Each treatment had three replications. The experiments started on November 30, 2016. The numbers of branches, nodes, floating leaves, and submerged leaves, and biomass weight of the plants in each container were measured on the week 0, 1, 2, 3, 4, 5, 6, 8, 10, 12, 16, 22, and 28.

### Endocide treatments

In each of the 17 plastic containers (14 × 15 × 5 cm, 0.68 L each), nine healthy and untreated living plants were cultured in tap water in the greenhouse. The nine plants included three plants of form B (approximately 1 g in fresh weight per plant), three plants of form E (approximately 2 g per plant), and three plants of form L (approximately 4 g per plant). Plants in each container were sprayed with 10 mL Nanopure™ water or an experimental endocide at various concentrations. The experiments started on December 17, 2015. Plant growth, morphological variation, and survival status were documented and photographed in each treatment for six weeks after the initial treatment. Then any newly developed plants in each container were transferred into a container with new water for 10 weeks of culture observations.

### Trichome damage experiments

A total of 72 healthy and untreated living plants of form L (approximately 5 g in fresh weight each) were cultured and tested in 24 plastic containers (14 × 15 × 5 cm, 0.68 L) with three plants in each container. The 24 containers of plants were randomly classified into eight groups with three contains each. Plants in each group received one of the following eight treatments. Control: the plants had no any treatment. Rubbing: the upper surfaces of the three pairs of leaves in each plant were rubbed by latex gloves with physical damage on trichomes only. Hole-punching: the three pairs of leaves in each plant were punched a hole (diameter: 0.635 cm) on each leaf blade by a hole punch. Trichome removal: the trichomes on upper leaf surfaces of three pairs of leaves in each plant were gently removed by a blade. Dyne-Amic® on lower leaf surface: three pairs of leaves of each plant were treated by 8 μL of 0.5% Dyne-Amic® on each of the lower blade surfaces by pipet. Dyne-Amic® on upper leaf surface: three pairs of leaves of each plant were treated by 8 μL of 0.5% Dyne-Amic® on each of the upper blade surfaces by pipet. Salvinicide III on lower leaf surface: three pairs of leaves of each plant were treated by 8 μL of 0.5% salvinicide III on each of the lower blade surfaces by pipet. Salvinicide III on upper leaf surface: three pairs of leaves of each plant were treated by 8 μL of 0.5% salvinicide III on each of the upper blade surfaces by pipet. The leaf surfaces of the experimental plants were checked and photographed daily for three weeks by a ×60 portable microscope linked to an iPhone.

### Data statistical analysis

The data were examined and few outliers that located outside three interquartile ranges were removed. The verified data were analyzed using the analysis of variance based on various linear models. For the density experiments, the model tested the effects of the form, density, week and their interactions on the numbers of branches, nodes, floating leaves, and submerged leaves and biomass weight. For the floating leaf folding experiment, the model tested the effects of the form, density, culture, day and interactions on the folding status. For the fragmenting experiment, the model included the form, treatment, week and their interactions. All factors were treated as fixed effects. A random error was added to each model. A repeated measures analysis used to account for the longitudinal nature of the data, specifically, using the SP (POW) structure for the density experiment and the fragmentation experiment (time intervals are not equal) and the AR(1) structure for the floating leaf folding experiment (time intervals are equal). LS means for the combinations of main factors were calculated by each time point and plots were made to show effects of main factors over time. Note that here and elsewhere in the text, except where otherwise indicated, the term significant refers to P < 0.05. All the analyses were performed using SAS^41^.

## Electronic supplementary material

Supporting Information
