# Supplementary material for: Endocide-Induced Abnormal Growth Forms of Invasive Giant Salvinia (Salvinia molesta)
Source: Sci Rep. 2018 May 22;8:8006. doi: 10.1038/s41598-018-25986-5 (PMC5964246; doi:10.1038/s41598-018-25986-5)
Supplement: Supplementary file 1 — Supporting Information [file 41598_2018_25986_MOESM1_ESM.pdf]

Supplementary Materials for

Endocide-Induced Abnormal Growth Forms of Invasive Giant Salvinia (*Salvinia molesta*)

Shiyou Li[1,*], Ping Wang[1], Zushang Su[1], Emily Lozano[1], Olivia LaMaster[1], Jason B. Grogan[1], Yuhui Weng[1], Thomas Decker[2], John Findeisen[2], and Monica McGarrity[2]

[1] National Center for Pharmaceutical Crops, Arthur Temple College of Forestry and Agriculture, Stephen F. Austin State University, Nacogdoches, Texas 75962, USA
[2] Inland Fisheries Division, Habitat Conservation Branch, Texas Parks and Wildlife Department, 4200 Smith School Rd., Austin, Texas 78744, USA
* Corresponding author: Tel.: +1 936 468 2071; fax: +1 936-468-2058. E-mail: lis@sfasu.edu

This PDF file includes:
Materials and Methods
Results
Supplementary Text
Figs S1 to S6
Table S1

1. Materials and methods

1.1. Plant Materials: Description of 12 Different Forms.

12 different forms of *S. molesta* have been observed in the greenhouse or the field by using the classification characteristics to distinguish the known three stages (forms) of *S. molesta*, (Fig. 1, Supplementary Table S1). Forms A and B (known as the primary stage (initialized I)) refers to the plants with small and flat floating leaves that are less than 15 mm in width. Form B refers to the plants with small and slightly folded floating leaves that are less than 15 mm in width. Form C refers to the plants with small and tightly folded floating leaves that are less than 15 mm in width when forced open. Form D refers to the developing plants with flat floating leaves that are between 15 to 50 mm in width. This form was not included in the experiments. Form E wide (known as the secondary stage (II)) refers to the developing plants with slightly cupped floating leaves that are between 15 and 50 mm in width. Form F refers to the developing plants with tightly folded floating leaves that are between 15 and 50 mm in width when forced open. Form G refers to the mature plants with sporocarps and slightly cupped floating leaves that are between 15 and 50 mm in width. Form H refers to the mature plants with sporocarps and tightly folded floating leaves that are between 15 and 50 mm in width when forced open. Form I refers to the developing plants with

slightly cupped floating leaves that are more than 50 mm in width. Form J refers to the developing plants with tightly floating leaves that are more than 50 mm in width when forced open. Form K refers to the mature plants with sporocarps and slightly cupped floating leaves more than 50 mm in width. Form L (known as the tertiary stage (III)) refers to the plant with sporocarps and tightly folded and large floating leaves that are more than 50 mm in width when forced open.

2. Results:

2.1. **Impact of population density on growth forms** (Supplementary Figures S1, S2, and S3).

2.2. **Impact of fragmenting on growth forms** (Supplementary Figures S4, S5, and S6).

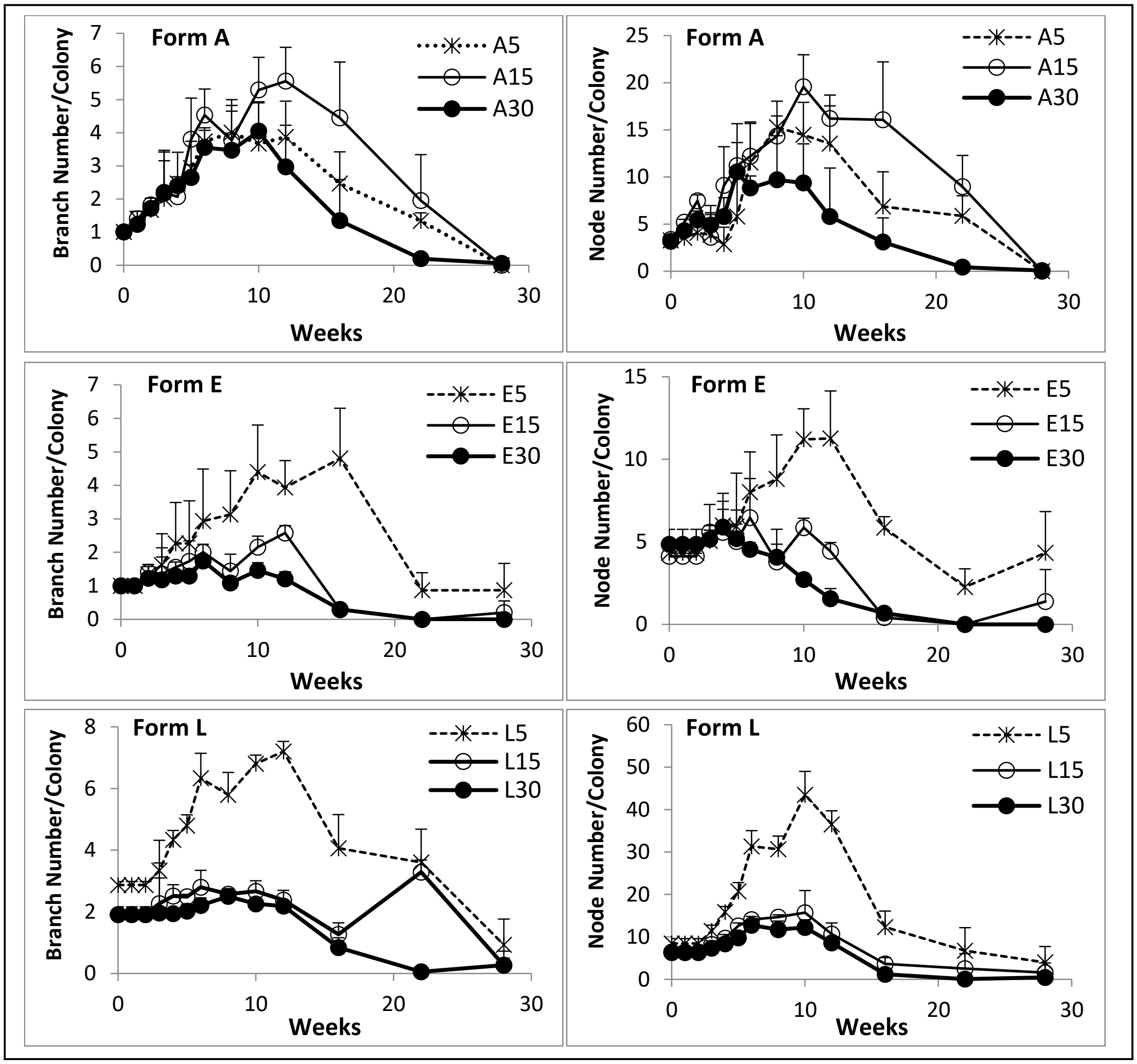

Supplementary Figure S1. Changes of branch and node numbers per plant of the forms A, E, and L at density of 5, 15, and 30 plants/container during the 28 weeks of experimentation.

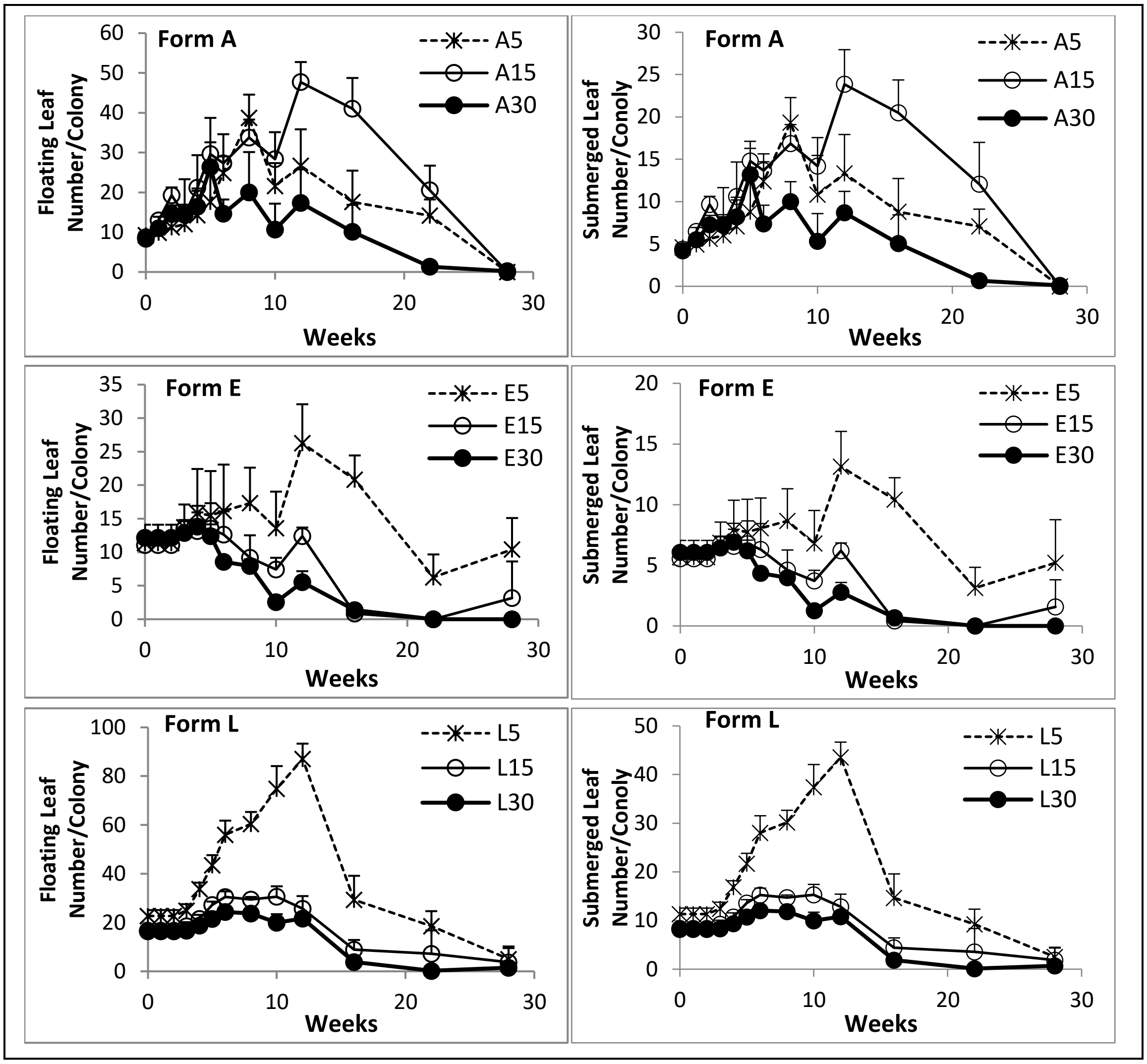

Supplementary Figure S2. Changes of floating and submerged leaf numbers per plant of the forms A, E, and L at density of 5, 15, and 30 plants/container during the 28 weeks of experimentation.

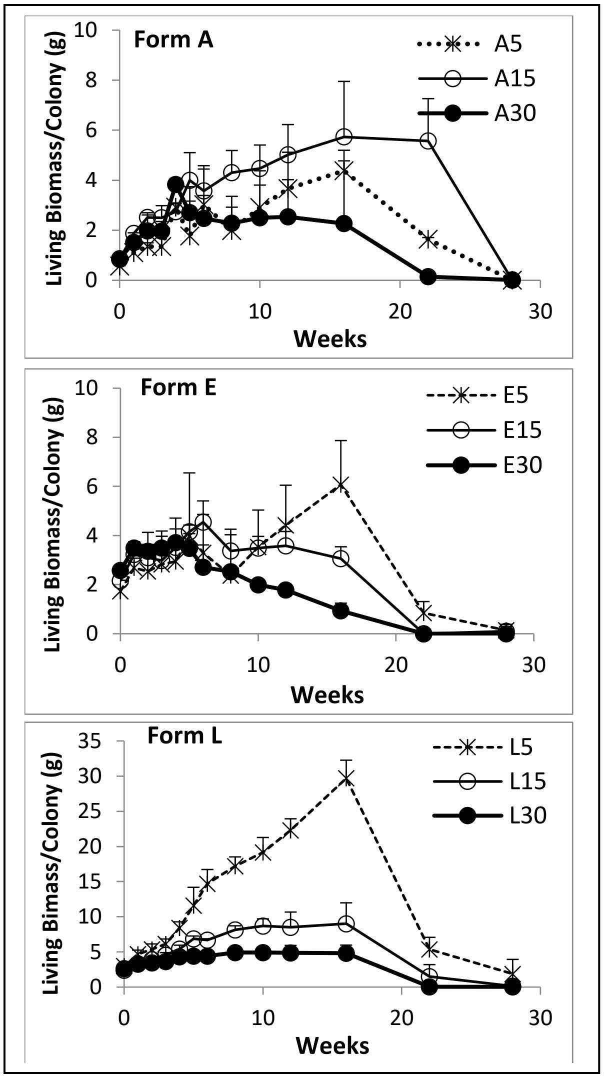

Supplementary Figure S3. Changes of total living biomass per plant of the forms A, E, and L at density of 5, 15, and 30 plants/container during the 28 weeks of experimentation.

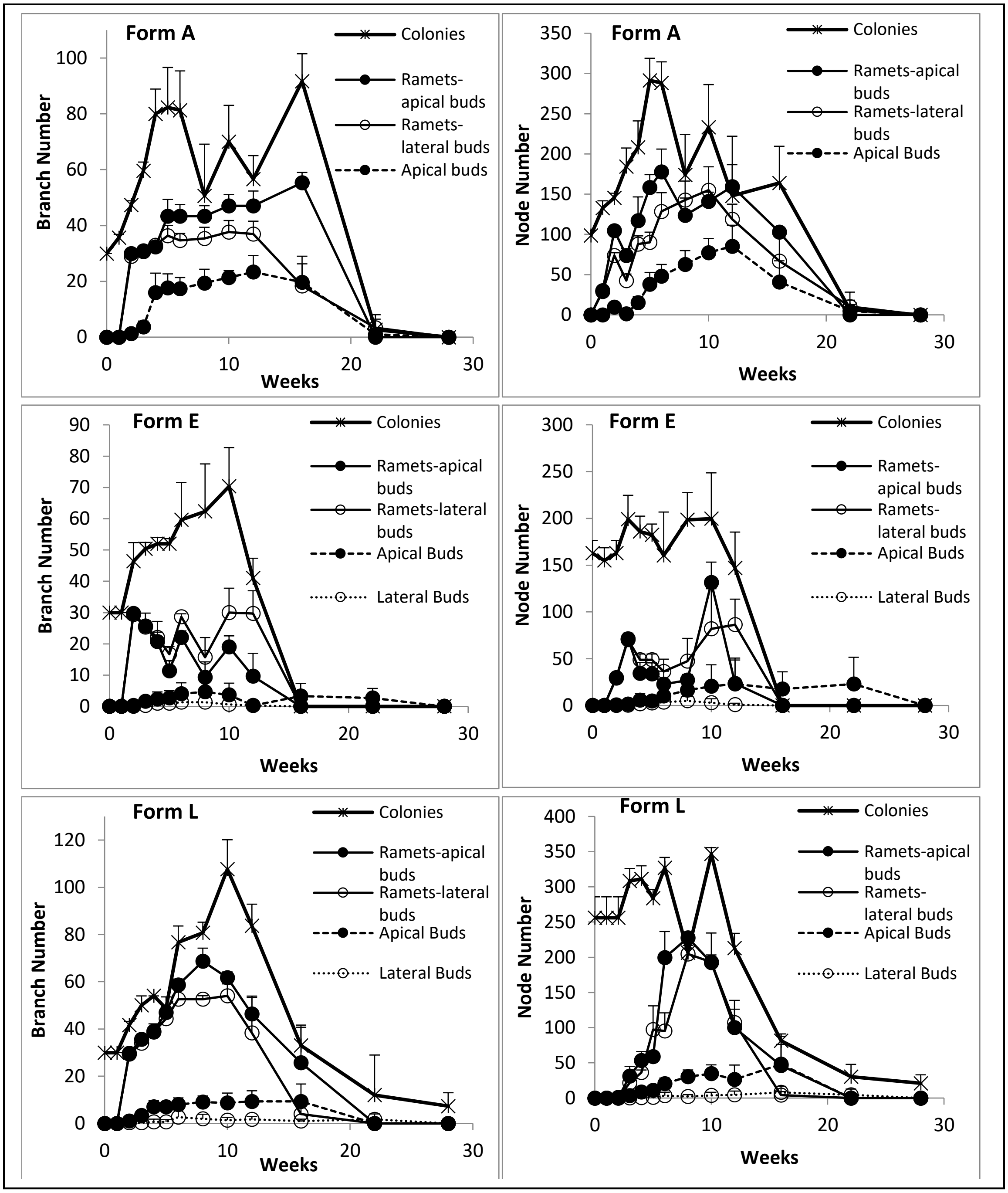

Supplementary Figure S4. Changes of branch and node numbers (per container) of colonies without fragmenting, ramets with apical buds, ramets with lateral buds, apical buds, and lateral buds of forms A, E, and L during the 28 weeks of experimentation.

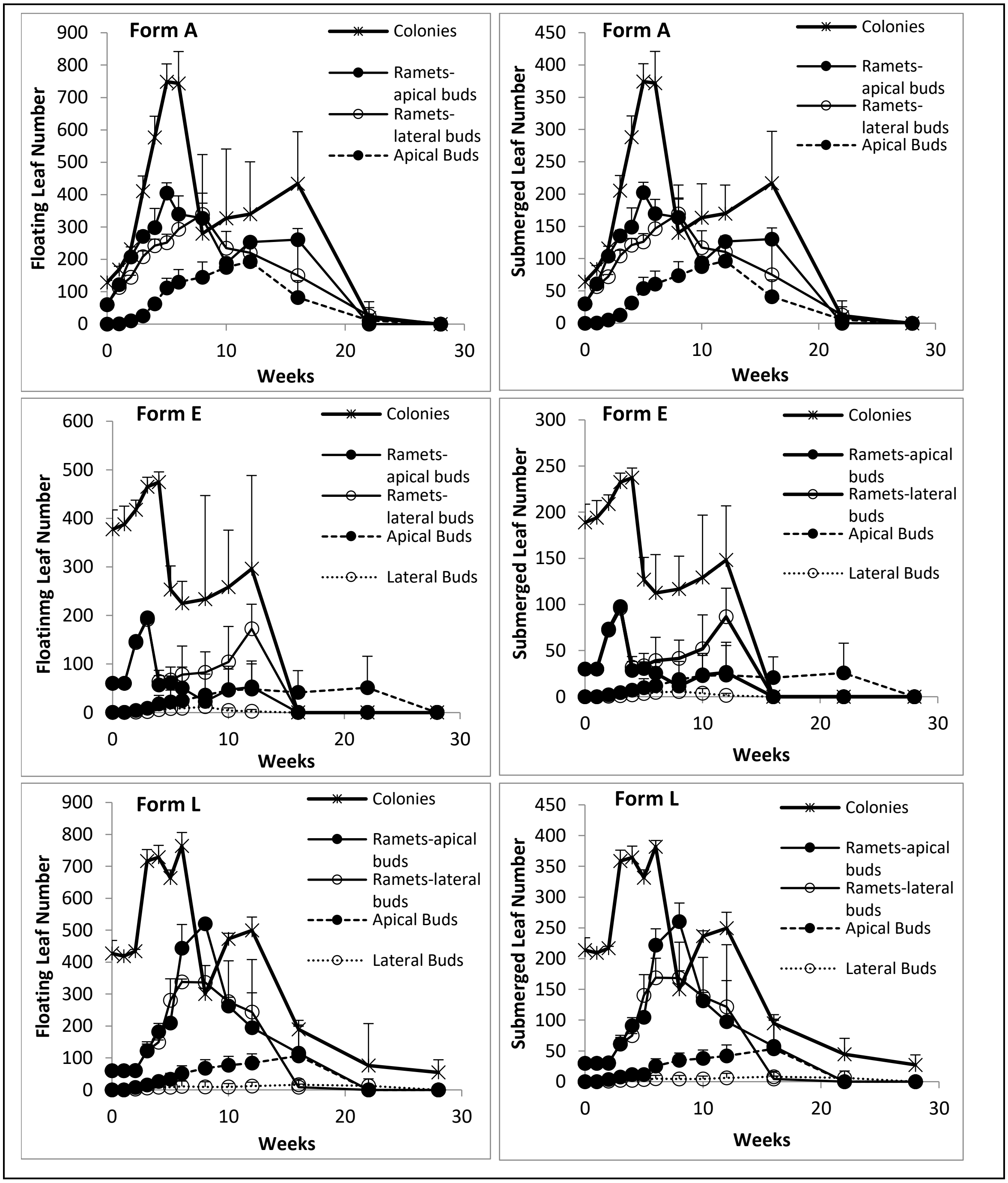

Supplementary Figure S5. Changes of floating and submerged leaf numbers (per container) of colonies without fragmenting, ramets with apical buds, ramets with lateral buds, apical buds, and lateral buds of forms A, E, and L during the 28 weeks of experimentation.

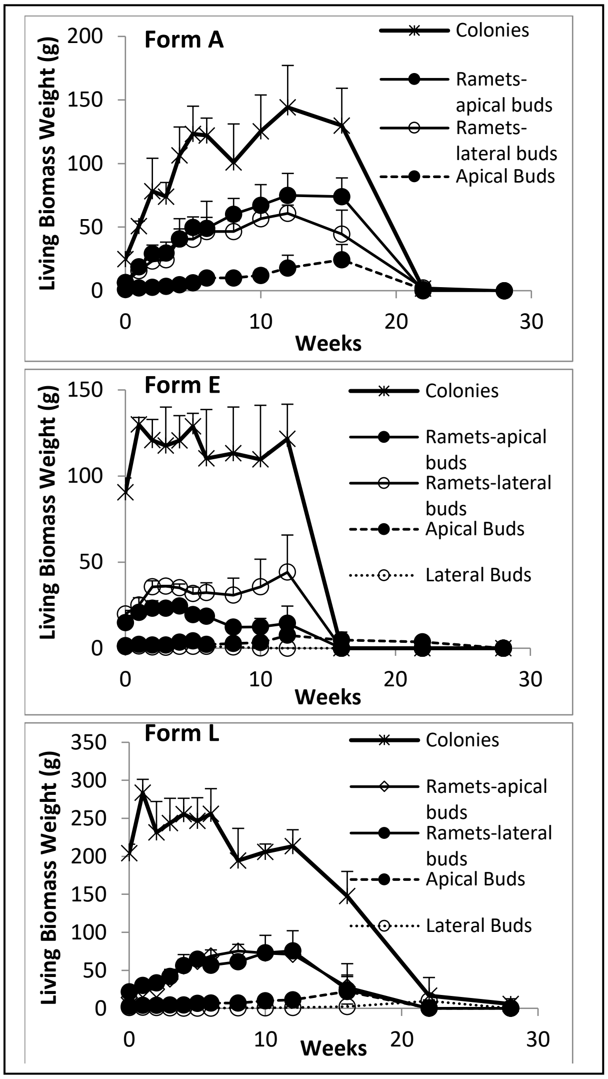

Supplementary Figure S6. Changes of total living biomass (per container) of colonies without fragmenting, ramets with apical buds, ramets with lateral buds, apical buds, and lateral buds of forms A, E, and L during the 28 weeks of experimentation.

Supplementary Table S1. Growth forms of *S. molesta* observed in the greenhouse or field investigations.

Growth Form	Width of Floating Leaves (fronds) When Forced Open (mm)			Open Status of Floating Leaves (fronds)			Sporocarps		Reported Growth Stage (phase or form)[21,29]	Observed Conditions
	**<15**	**15-50**	**>50**	**Flat**	**Slightly cupped**	**Tightly folded**	**no**	**yes**		
**A**	+			+			+		I	A small abnormal form is produced when adequate space is available after fatal disturbances*
**B**	+				+		+			A small abnormal form produced when limited space is available after fatal disturbances*
**C**	+					+	+			A small abnormal form is produced under crowded conditions after fatal disturbances*
**D**		+		+			+			A medium-size immature normal form is developed from some abnormal forms or new growth of other normal forms when adequate space is available
**E**		+			+		+		II	A medium-size immature normal form is developed from some abnormal types or new growth of other normal forms when limited space is available
**F**		+				+	+			A smaller immature normal form is developed from some abnormal forms or new growth of other normal forms under crowded conditions
**G**		+			+			+		A medium-size mature normal form is developed from

										form D or E when limited space is available
**H**		+				+		+		A medium-size mature normal form is developed from form F under crowded conditions
**I**			+		+		+			A large immature normal form is developed from form D or E
**J**			+			+	+			A large immature normal form is developed from form D, E, or F under crowded conditions
**K**			+		+			+		A large mature normal form is developed from other normal forms when limited space is available
**L**			+			+		+	III	A large mature normal form is developed from other normal forms under crowded conditions

* Fatal disturbances such as extensive herbicide, weevil, or endocide treatments, physical damage, extreme climatic conditions (e.g., drought and freeze).
